# Psychophysical evidence for the number sense

**DOI:** 10.1098/rstb.2017.0045

**Published:** 2018-01-01

**Authors:** David C. Burr, Giovanni Anobile, Roberto Arrighi

**Affiliations:** 1 Department of Neuroscience, Psychology, Pharmacology and Child Health, University of Florence, Florence, Italy; 2 School of Psychology, University of Sydney, Sydney, Australia; 3 Department of Translational Research on New Technologies in Medicines and Surgery, University of Pisa, Pisa, Italy; 4 Department of Developmental Neuroscience, Stella Maris Scientific Institute, Calambrone, Pisa, Italy

**Keywords:** numerosity, texture density, numerical cognition, approximate number system, subitizing

## Abstract

It is now clear that most animals, including humans, possess an ability to rapidly estimate number. Some have questioned whether this ability arises from dedicated numerosity mechanisms, or is derived indirectly from judgements of density or other attributes. We describe a series of psychophysical experiments, largely using adaptation techniques, which demonstrate clearly the existence of a number sense in humans. The number sense is truly general, extending over space, time and sensory modality, and is closely linked with action. We further show that when multiple cues are present, numerosity emerges as the natural dimension for discrimination. However, when element density increases past a certain level, the elements become too crowded to parse, and the scene is perceived as a texture rather than array of elements. The two different regimes are psychophysically discriminable in that they follow distinct psychophysical laws, and show different dependencies on eccentricity, luminance levels and effects of perceptual grouping. The distinction is important, as the ability to discriminate numerosity, but not texture, correlates with formal maths skills.

This article is part of the discussion meeting issue ‘The origins of numerical abilities’.

## 1. Introduction

Although humans may be the only species with a linguistically mediated code for numbers, we share an approximate, non-verbal representation of number with many animal species, as many papers in this special issue make amply clear. The evolutionary advantage of this capacity is obvious, facilitating choice of areas with more food, and/or more conspecies, and allowing quick determination of which group of competitors is more numerous. But how is numerosity sensed—directly, by a *number sense*, as suggested by the physiological and behavioural studies in many species [1–6], or indirectly via other means, such as estimating texture density [7–12]? What are the limits of the *number sense*?

## 2. Adaptation and number

One clear signature of the existence of a dedicated perceptual mechanism is its susceptibility to adaptation [13–15]: several seconds of exposure to a given stimulus—say leftward motion—changes the appearance of subsequent stimuli viewed in the same position, causing a negative aftereffect, illusory rightward motion in this case [16]. Adaptation is ubiquitous throughout all sensory systems. It is a form of experience-dependent plasticity, probably serving, at least in some cases, an active functional role, such as calibrating perceptual systems to their environment by dynamically tuning responses to match the distribution of stimuli to make maximal use out of the limited working range of the system [17–20] (although this is clearly not the only role [21,22]). Traditionally, adaptation has been used by psychophysicists to reveal neural mechanisms selective to specific aspects of the stimulus, such as direction of motion [23] or orientation [24].

Burr & Ross [25] recently showed that numerosity is strongly susceptible to visual adaptation. The effect is illustrated in the online animation (electronic supplementary material, movie S1): after a period of observing dense or sparse dot clouds (approx. 30 s), the apparent numerosity of subsequently viewed dot clouds changes considerably. The adaptation effects are spatially specific, so it is possible to simultaneously adapt different locations of the visual field to high, low or neutral numerosities [26].

**Figure 1.**
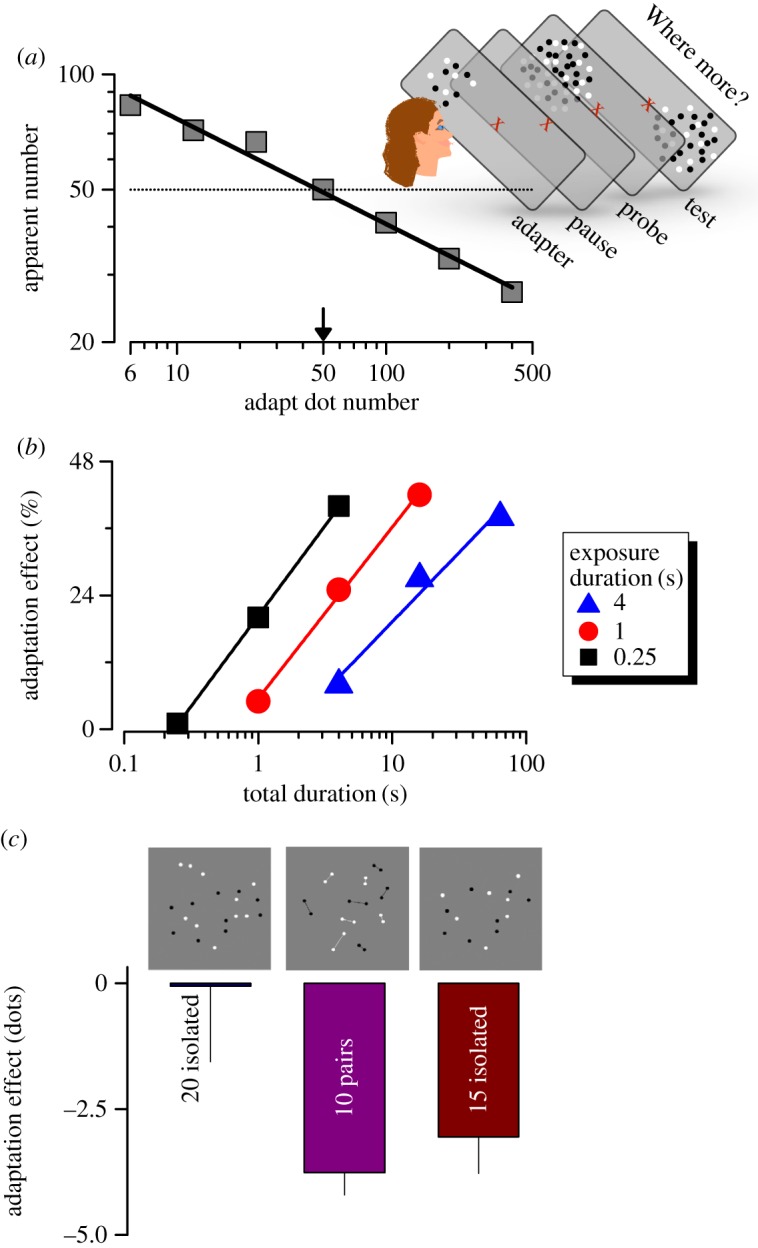
Adaptation to the numerosity of spatial arrays of dots. (*a*) Effect of adaptation as a function of adaptor numerosity. Subjects adapted to dot patterns of numerosities ranging from 5 to 500, then tested the adaptation effect by comparing a test stimulus presented at the adaptation site with a 50-dot probe stimulus presented at a different location. The perceived numerosity of the test increases after adaptation to low numerosities, and decreases after adaptation to high. (*b*) Effect of exposure duration and repetition. Adapting stimuli of duration 0.25 s (black symbols), 1 s (red symbols) or 4 s (blue symbols) were presented 1, 4 or 16 times before the test was displayed (successive symbols of each colour). The ordinate plots the adaptation effect, calculated as the percentage change in apparent numerosity. Exposure duration has little effect on the magnitude of adaptation, only the number of presentations. (*c*) Effect of adapting to a dot pattern of 20 unconnected dots on: 20 unconnected dots (left); 20 connected dots (centre); 15 unconnected dots. Adaptation clearly operates on apparent, not physical numerosity. Reproduced with permission from Burr & Ross [25], Aagten-Murphy & Burr [26] and Fornaciai *et al*. [27].

Interestingly, the demonstration does not work well presented on paper, asking subjects to change gaze from adaptor to test. This suggests that the effect is not completely retinotopic. Perhaps both adaptor and test need to be in the same *spatiotopic* position, the same position on the screen, not the retina, as we describe later for adaptation to temporal stimuli, and are currently studying for spatial stimuli. Figure 1*a* shows the effect of adapting to different numerosities on the apparent numerosity of a 50-dot display. Adapting to higher numerosities caused subjects to underestimate the apparent numerosity of the display and, most interestingly, adapting to low numerosities caused an overestimation, while adapting to 50 had no effect at all. The effects are large, up to a factor of two in each direction.

Adaptation to number is fascinating on several levels. The fact that the apparent numerosity of the same physical cloud of dots can vary so greatly after simply observing a different dot cloud is a clear demonstration that we do not actually encode each individual dot. After adaptation to high numerosities, no particular dot *disappears* from the test patch; and after adaptation to low numerosities, new dots are not *created*. Clearly, the system does not encode all dots individually, but creates an efficient description of the scene, perhaps just the numerosity and some other simple summary statistics (consistent with much evidence that the perceived richness of world is very much an illusion [28]).

The temporal dynamics of numerosity adaptation are interesting [26]. Figure 1*b* shows how the magnitude of the effect depends on repeated exposure to stimuli, for various durations of each exposure. As is to be expected, the more often the adaptation stimuli are presented, the greater the effect. However, the magnitude of the effect does not depend on the duration of the exposure to adaptation: the effects for stimuli of 0.25, 1 and 4 s exposure were almost identical. This event-based numerosity fits well with statistical models of adaptation in which the dynamic adjustment of perceptual experiences, based on both the previous experience of the stimuli and the current percept, acts to optimize the limited working range of perception, implicating a highly plastic mechanism for numerosity perception dependent on the number of discrete adaptation events [29].

The fact that brief periods of adaptation were sufficient to elicit large changes in apparent numerosity allowed us to study the neural effects of adaptation, using functional magnetic resonance imaging (fMRI) techniques. Castaldi *et al*. [30] recorded the BOLD responses to various numerosities from intraparietal sulcus (IPS) and V1 in human observers, before and after they had adapted to an 80-dot stimulus. The authors trained a classifier to discriminate the numerosity of dot clouds before and after adaptation. The classifier worked well in IPS but not V1, because the overall energy of the stimuli was balanced across numbers. Importantly, IPS classifiers trained with pre-adaptation presentations could accurately decode number only from other pre-adaptation trials, and not from post-adaptation presentations, and vice versa. This suggests that adaptation changes the cortical maps underlying the representation of numerosity in IPS, not at early stages of analysis (as some have suggested [8]).

A major theme of this review is whether human vision is endowed with a dedicated number sense, or whether numerosity discrimination acts via other visual mechanisms, such as density analysis. One of the earliest and perhaps clearest demonstrations that number is not driven by density alone was discovered independently by Franconeri *et al*. [31] and He *et al*. [32], illustrated in figure 1*c*. The two left-most stimuli in the figure 1*c* both comprise 20 randomly distributed dots, but in the central figure pairs of dots have been joined. The impression, especially for brief presentations (see also electronic supplementary material, movie S2), is that the numerosity of the connected pattern is considerably lower than that of the unconnected dots. When measured formally, the pattern with 20 connected dots appears to comprise only 15 dots, like that on the right [27]. Why should the connected-dot patterns appear less numerous? If numerosity were based, even partially, on texture density, adding lines to the pattern should increase numerosity, as it clearly increases the amount of ‘stuff’ in the pattern (see also figure 5). Presumably connecting the dots with a line perceptually links them into a single unit, reducing the estimate of numerosity, which seems to be based more on the number of separable items, rather than how much stuff is in the field of view.

Not only do connected-dot patterns *appear* to be less numerous, but imaging studies show that IPS encodes them as having lower numerosity, consistent with their perceived rather than physical numerosity [33]. Given this evidence, we asked whether adaptation would also operate on perceived rather than physical numerosity [27]. Adapting to the same numerosity as the test does not change the numerosity ([25,30], figure 1*a*). So after adapting to 20 dots, viewing 20 isolated dots is veridical, with no bias (figure 1*c*). However, adapting to the same 20-dot pattern does affect the apparent numerosity of 20 connected dots, causing the pattern to appear to have four fewer dots, on average. This is the same magnitude of bias as observed with a 15-dot test, which matches the apparent numerosity of the 20-connected-dot pattern. Thus, it would seem that adaptation operates on the apparent numerosity rather than on the physical numerosity, consistent with the idea that adaptation occurs at a reasonably high level, probably IPS, as the fMRI studies [30] suggest.

## 3. Numerosity of temporal sequences

**Figure 2.**
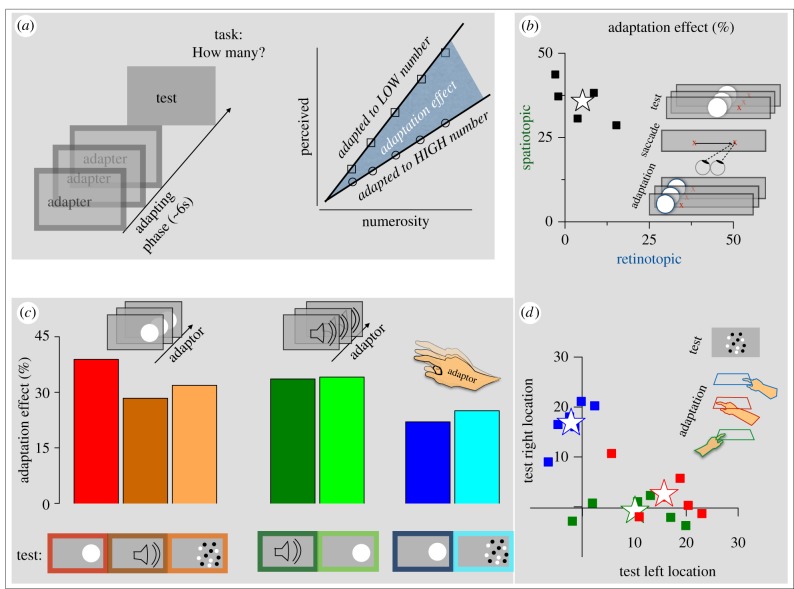
Adaptation to temporal sequences. (*a*) Subjects adapted to pseudo-random sequences of stimuli (flashes or tones) averaging 2 or 8 stimuli per second, then estimated the apparent numerosity of a test (sequences of flashes, tones, or spatial arrays). The effect of adaptation was multiplicative, with adaptation to slow and fast stimuli increasing or decreasing the perceived numerosity, respectively. The adaptation effect was taken as the difference of the best fitting regression lines constrained to pass through zero (black lines bounding the dark-shaded region). (*b*) Adaptation to sequences of temporal events is selective in spatiotopic, not retinotopic coordinates. Black squares plot the magnitude of spatiotopic adaptation against the retinotopic adaptation for individual subjects, who made a saccade between adaptation and test. Adaptation occurred only if the test and adaptor were in the same spatiotopic position (same position on screen), not if they were in the same retinotopic location (as illustrated in the icon). The star plots averaged results. (*c*) Magnitude of the adaptation effect for various conditions: adapting to sequences of flashes and testing with sequences of flashes (red), tones (brown) or spatial arrays (orange); adapting to sequences of sounds and testing with sequences of sounds (dark green) or flashes (light green); adapting to fast and slow tapping and testing with sequences of flashes (darker blue) or spatial arrays (light blue). (*d*) Adaptation to motor events is selective in spatial, not hand coordinates. The colour-coded squares plot for individual subjects adaptation magnitude after fast and slow tapping for stimuli presented to the right side of the screen against those presented to the left side. The tapping hand was either the left or the right, tapping on the left or right side (as indicated by the colour-coded icons). Whichever the hand, the tapping must be on the same spatial side as the test for there to be adaptation. Stars show averaged results. Reproduced with permission from Arrighi *et al*. [35] and Anobile *et al*. [36–38].

Humans are capable of estimating the number of items not only in spatial patterns, but also in sequences of events over time. As mentioned in another publication in this issue, Nieder *et al*. [34] have described neurons in monkey pre-frontal cortex that respond similarly both to temporal sequences of events and to spatial arrays of matched numerosity. We have recently used adaptation to demonstrate the existence of mechanisms in human brain selective for the numerosity of temporal sequences [35]. We presented sequences of briefly displayed discs of light, and asked subjects to estimate their number, both before and after adapting to slow (2 flashes s^−1^) or fast (8 flashes s^−1^) sequences (figure 2*a*). As with spatial adaption, adapting to slow sequences caused a subsequently displayed sequence to appear more numerous, while adapting to fast sequences caused the sequence to appear less numerous. The adaptation effect was multiplicative, reducing or increasing perceived numerosity by a scale factor, thereby changing the slope of the regression line relating perceived to physical number (figure 2*a*). We defined the magnitude of the adaptation effect as the difference in the slopes of the regression lines, after adapting to high and low rates of sequences (shaded area of figure 2*a*).

Like adaptation to spatial numerosity, the temporal numerosity aftereffect was spatially selective. Adaptation occurred only when the test sequence was displayed at the same position as the adaptor sequence: when the test was displayed on the opposite side of the screen compared with the adaptor, there was very little effect of adaptation. This suggests that the adaptation is a perceptual rather than a cognitive phenomenon, such as internal counting. Importantly, the spatial selectivity had to be in external not retinal coordinates for the adaptation to be effective. In the data shown in figure 2*b*, subjects made a saccadic eye movement between the adaptation and test phases, and the test was then displayed either in the same position in space as the adaptor or the same position on the retina (as in the icon of figure 2*b*). Only when displayed at the same position on the screen (ordinate of scatterplot) was there a strong adaptation effect: displayed at the same retinotopic position in the same experiment caused very little adaptation (abscissa). That the adaptation was spatiotopic, not retinotopic, suggests it occurs at a reasonably high level of information processing, as has been observed for event duration [39] and the positional motion after effect [40,41]; but see also [42].

Working in time rather than space lends itself well to cross-modal studies, particularly with audition, which has good temporal but weak spatial resolution. Our experiments showed that adaptation to temporal sequences also occurs with tones. Furthermore, adapting to a series of tones changed the apparent numerosity of visual flashes, and vice versa, to the same extent as within-modal adaptation (figure 2*c*; electronic supplementary material, movies S3 and S4), pointing to the existence of a number sense that transcends sensory modality. Perhaps even more surprising was the ‘cross-format’ adaptation: adapting to a series of centrally displayed flashes changed the apparent numerosity of a spatial array of dots, by a similar amount to the purely temporal adaptation (figure 2*c*).

All these results point to the existence of a very generalized number sense, transcending space and time, and sensory modality. As numerosity can be important for the generation of actions, and there is neurophysiological evidence linking them [43,44], we asked whether adapting to actions could affect number perception. Subjects tapped their fingers in mid-air either rapidly or slowly, then judged the numerosity of sequences of flashes, or of arrays of dots. As with adaptation to sequences of flashes, adapting to slow tapping caused overestimation and adapting to fast sequences underestimation [36]. Again, adaptation works equally well both for sequences of flashes and for clouds of dots (figure 2*c*). Adaptation to action also affects the apparent numerosity of auditory sequences [45].

Figure 2*d* shows that, like adaptation to sequences of stimuli, adaptation to tapping is spatially selective. And just as the temporal adaptation is selective in spatiotopic rather than retinotopic coordinates, adaptation to tapping is selective to the spatial position of the tapping hand, not to which hand does the tapping. Under the three conditions tested—right hand tapping right and left, and left hand tapping left—the adaptation effects were strong only when the hand (either left or right) was tapped on the same side as the stimuli were presented.

## 4. Spontaneous detection of numerosity

There has been a good deal of discussion from psychophysicists about whether numerosity is sensed directly by vision, or whether it is sensed indirectly via texture mechanisms tuned to density [7–11,25,37]. We have already described one example suggesting that this is unlikely: connecting pairs in dot patterns causes them to appear less numerous (even though the added lines increase their density), and this also affects adaptation (figure 1*c*).

**Figure 3.**
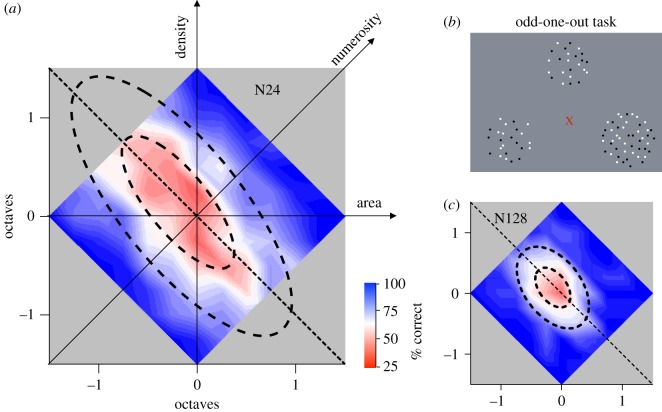
Spontaneous discrimination of numerosity. (*a*) Two-dimensional psychometric functions on a space spanned by log-area and log-density, for sparse standard stimuli (24 dots, 40 deg^2^, 0.6 dots deg^−2^). The heat map refers to the interpolated per cent correct for discriminating various points sampled over the coloured area from the origin (with 33% chance guessing rate). The ellipses are fitted to pass through 50 and 75% correct judgement. (*b*) Illustration of the stimuli used in the odd-one-out task. Two were identical, defined by the origin of the space (24 dots, 40 deg^2^). The target (lower right) was taken from many different sample points in the space (in this case, 48 dots, 80 deg^2^). (*c*) The same as (*a*), but for denser standard stimuli (128 dots, 40 deg^2^, 3.2 dots deg^−2^). Reproduced with permission from Cicchini *et al*. [46].

Nevertheless, it is reasonable to ask what is the natural dimension for spontaneously discriminating patterns that vary in quantity and area: numerosity or density, or some combination of both? Cicchini *et al.* [46] tackled this question directly, following a technique borrowed from colour science. They measured for numerosity the equivalent of ‘MacAdam ellipses’ [47], ellipses in colour space inside which all stimuli are indiscriminable. The short axes of these ellipses indicate the most sensitive direction in that space, pointing to the existence of specific mechanisms (see also [48]). Numerosity is the product of density and area, so the logarithm of numerosity is the sum of log-area and log-density. Thus, there exists a two-dimensional ‘numerosity space’, spanned by log-area and log-density, with log-numerosity following the positive diagonal (figure 3*a*). Cicchini and co-workers [46] measured discrimination thresholds within this space, using an ‘odd-one-out’ technique (figure 3*b*), where subjects had to identify the target from two standards. The two standards were defined by the origin of the space (24 dots: 0.6 dots deg^−1^, 40 deg^2^), while the target was selected at random from various points distributed throughout the coloured diamond. The two-dimensional psychometric functions are well described by a highly elongated ellipse, whose short axis, defining maximum sensitivity, is clearly aligned to the numerosity diagonal. Sensitivity along this axis was six times higher than in the orthogonal direction, showing that numerosity is the most sensitive dimension: just as red–green is a sensitive direction in colour space.

That numerosity emerged spontaneously when subjects were given no specific instructions, but only asked to identify the odd one out, tells a good deal of what is the more natural cue for discrimination of quantity. However, Cicchini *et al*. [46] also asked subjects to make explicit judgements about stimuli within the numerosity space, judging—in separate sessions—whether the test stimulus appeared to be more numerous, denser or of greater area, than the standard. The results were interesting. The discrimination boundaries for these judgements should be oriented at 45, 0 and 90° for number, density and area, respectively, if the judgements were really based on that particular property. The data showed that the boundary for number judgements was oriented at 37°, biased slightly away from 45°, towards the area axis and away from density (by about 17%), agreeing with other studies (e.g. [7,49]). However, the boundary for area judgements was 66° rather than 90°, a massive shift of 53% towards number, suggesting that number was as important as area in making area judgements; and the boundary for density was 35° rather than 0°, shifted towards number by 78%, suggesting that density judgements are mediated almost entirely by numerosity (rather than the other way round). These results are not at odds with those suggesting that number estimates can be slightly influenced by area or density: these small effects do occur. However, the interactions in the other direction are in fact far stronger: number strongly influences both area and density, suggesting that number is the more robust and basic attribute.

## 5. The relationship between numerosity and texture density

Although there exists good evidence that numerosity is perceived independently of texture, common sense suggests that the two must be related in some way. If we continue to increase the number of items within a given area, we will reach a point where we can no longer resolve the items, and they will merge into what is commonly termed *texture*. When does this occur? What determines whether an array of items turns into an amorphous texture?

**Figure 4.**
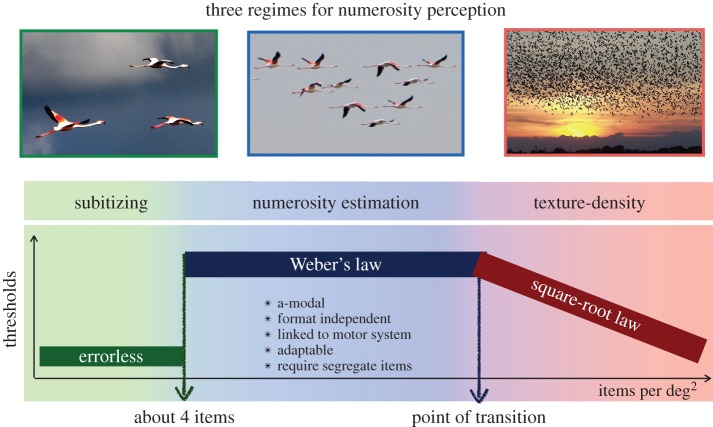
Illustration of the three regimes of numerosity perception: subitizing, estimation and texture. Reproduced with permission from Anobile *et al*. [37].

In a previous review on this topic [37], we suggested that there exist three different regimes in number analysis (figure 4). First there is the well-described subitizing range, where judgements are fast and errorless, which extends up to about four items [50]. Then there is a range where items are discernable as unique from each other, and we can estimate their quantity, rapidly but with error (often referred to as the ‘approximate number system’). Finally, the density becomes too great to segment the items from each other, and the stimuli become textures. Initial evidence for this idea came from simple measurements of Weber fractions—the minimal detectable change in numerosity, normalized by point of subjective equality—over a large range of numerosities and densities [51]. Although it is commonly assumed that Weber fractions for numerosity are constant [28,52,53], when carefully measured over a wide range, it is clear that this is not strictly true. Weber's law holds for a while, then after a critical point, Weber fractions decrease at a rate proportional to the square root of numerosity. The critical numerosity is lower for smaller than for larger patches, corresponding to a critical density of about 0.3 dots deg^−2^: for example, 30 dots within 10 × 10 deg^2^. The existence of the two regimes—Weber's law and the square root law—is suggestive (but by no means proof) of the existence of two separate perceptual mechanisms. When the experiments are repeated with test and probe patches of different sizes (so numerosity is not directly proportional to density), these predictions hold over an even wider range, extending past the boundary. These experiments with stimuli of mismatched area suggest that the two regimes are not mutually exclusive, but overlap considerably. When the areas are matched and density can be used as a proxy for number, or vice versa, the more sensitive prevails; but the fact that the Weber and square root laws extend past the boundaries shows that there is considerable overlap in the mechanisms. Similarly, other experiments have shown that the estimation range extends into the subitizing range, revealed under conditions of divided attention, which selectively impair subitizing [54–56]. Piazza *et al.* [57] also report evidence for a strong overlap between subitizing and estimation.

We believe that the transition from numerosity to texture occurs when the individual items are no longer discernable as separate items, but blend into an amorphous mosaic. In other words, the items become ‘crowded’. Crowding is a well-studied visual phenomenon, referring to the fact that stimuli, typically letters, that are easily discerned when displayed individually, can become indiscriminable when embedded in other letters [58]. It is possible that a similar process governs the transition from numerosity to density. If crowding-like mechanisms are at play, we can make two clear predictions: the transition between numerosity should depend on eccentricity, occurring at lower densities in the periphery; and it should depend on centre-to-centre spacing rather than edge-to-edge separation, or total coverage of dots. Both these predictions were verified by recent data [59]: texture mechanisms came into play far earlier in the periphery than with central vision, and the effects did not depend on stimulus size. For numerosity mechanisms to operate, the items to be enumerated need to be perceptually segregated.

A good deal of other evidence has supported the concept of different mechanisms operating on sparse and dense patterns. For example, Ross & Burr [60] showed that numerosity depended on luminance, with apparent numerosity increasing with decreasing luminance, by 6% per log-unit, while dense textures were entirely unaffected by luminance. Cicchini and co-workers [46] also repeated the experiment described in figure 3 with dense patterns of 128 dots, corresponding to 3.2 dots deg^−2^ (figure 3*c*). At these densities, the ellipse became much rounder, with an aspect ratio of 1.8 instead of 6. Indeed, the data of figure 3*c* are not inconsistent with the hypothesis that numerosity is derived from independent estimates of density and area. Besides supporting the notion of separate numerosity and texture mechanisms at different densities, the fact that the ellipse is quite different under these conditions shows clearly that the narrow ellipse for the modest density condition was not the only possible outcome, reinforcing the notion that specific numerosity mechanisms are at work. Interestingly, there is also support for the existence of three mechanisms in the older literature. Mandler & Shebo [61] reported three regimes of reactions times: an initial range of constant reaction times, one to three elements (subitizing), followed by a sharp increase in reaction times with item number, up to about seven items, followed by another plateau. The region of transition for the second plateau does not correspond exactly to what we observe, but the pattern of results is very similar.

**Figure 5.**
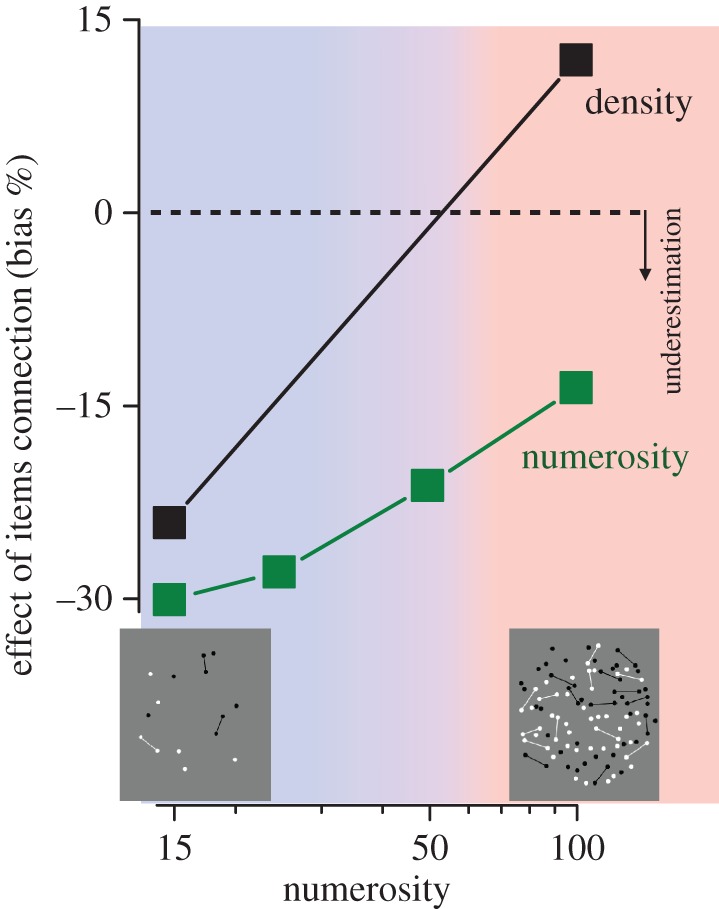
The effect of linking 40% of dots on apparent numerosity (green symbols) and apparent density (black symbols), as a function numerosity of the pattern. Reproduced with permission from Anobile *et al*. [62].

Another prediction we can make involves the connected patterns used in the experiment described in figure 1*c*. If the underestimation of numerosity of connected patterns relies on reducing the number of perceived entities, then the dots and the lines should be segmentable for this to work. In other words, we expect the connectedness effect to disappear or be greatly reduced at high densities. Figure 5 shows that this does occur [62]. At modest numerosities, connecting 40% of dots led to a 30% reduction in apparent number, agreeing with previous work [31,32]. This is interesting, as the Fourier transforms show that the connected patterns contain more energy at high spatial frequencies which, according to the influential model of Dakin *et al.* [7], should lead to an increase, rather than a decrease in perceived numerosity. However, when we measured the apparent numerosities of higher densities, the effect was reduced, being only 15% with of 100 dots (3.3 dots deg^−2^).

We also asked subjects to judge the density of the patterns. At low densities, the connected patterns seemed about 25% less dense, despite the fact that they were in fact more dense, as they have more patterning within the same area. However, at higher densities, the results inverted, with the connected patterns appearing to be denser, corresponding to the physical reality. Thus, at modest densities, it would seem that perceived density was driven by perceived numerosity, rather than the other way round, in agreement with other work [37]. At high densities, on the other hand, perception corresponds much more closely to physical reality.

Other evidence reinforces the idea of different regimes for numerosity and density perception at different stimulus densities. For example, Dakin *et al*. [7] have shown that stimuli occupying a larger area can appear more numerous than those of smaller area. However, this effect occurs only at high densities, within the texture regime: it disappears completely at moderate densities (see fig. 8 in [37]). Similarly, Zimmermann & Fink [63] studied numerosity after manipulating stimulus area with a size-adaptation technique [64]. Size-adaptation affected not only the area of the stimulus, but also the apparent numerosity of the dots within that stimulus. Importantly, the numerosity effects occurred principally at high densities, where texture mechanisms may be expected to operate: at lower numerosities, there was little effect.

### (a) Prediction of mathematical performance

**Figure 6.**
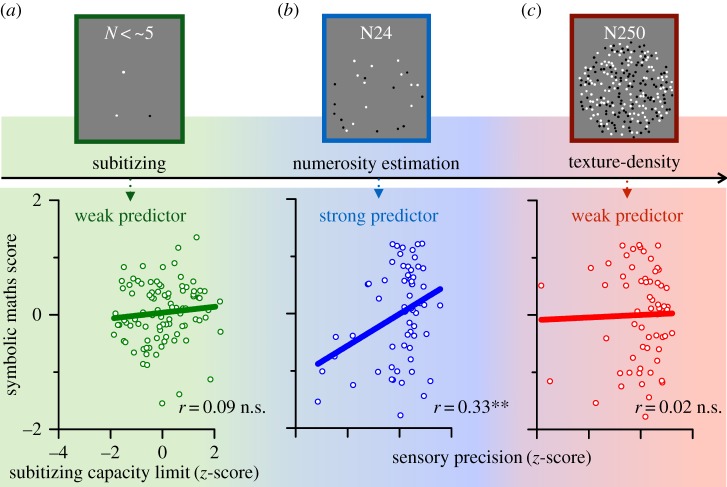
(*a*) Subitizing, (*b*) estimation and (*c*) density discrimination as predictors of maths ability. Only discrimination of modest numerosities (24-dot patterns) predicted maths scores significantly (*r* = 33, *p* < 0.01). Neither estimates of the subitizing limit nor density discrimination correlated significantly. Reproduced with permission from Anobile *et al*. [37,38,74].

A good deal of evidence shows that numerosity discrimination thresholds are a reliable predictor of both current and future maths achievements in school-age children [38,65–68], leading to the suggestion that numerosity perception may act as a form of ‘start-up tool’ for the acquisition of mathematical skills [68]. This is important, as it suggests that understanding the mechanisms behind numerosity may have practical implications. It also leads to a further prediction: that thresholds over the estimation range should correlate with maths scores, as previously reported by many, but over the texture density range, they should not. This is exactly what Anobile *et al.* [38] found. Figure 6 shows that for modest densities (24 dots), precision in numerosity discrimination predicted maths scores well, with a correlation of *r* = 0.33, whereas the correlation for the higher density (250 dots) was only 0.02 and not significantly different from zero. This finding is consistent with that of Tibber *et al.* [11], who also reported that numerosity but not density thresholds predict maths scores in their large sample of 300 children and adults. All these results are consistent with the notion that texture and numerosity are analysed by different mechanisms, and are important in showing that only numerosity thresholds predict maths abilities. Similarly, thresholds for disc size [69,70], visual motion velocity [38], auditory duration or line length [71] discrimination do not relate to maths skills.

Figure 6 also shows the scatterplot relating the subitizing limit (calculated using the technique described in [72,73]) to maths scores. Again the correlation was weak (*r* = 0.09), and non-significant [74]. We also correlated maths scores against precision for estimating temporal numerosities, for both visual and auditory stimuli, in the same cohort of children [74]. Also in this case, the correlations were not significant, although the temporal thresholds did correlate with the spatial estimation thresholds. This result is surprising, given the evidence for a generalized number sense summarized in figure 2. Perhaps, the development of mathematics is based primarily on the spatial mechanisms of numerosity perception, in line with ideas of ‘cultural recycling’ of the visual cortex for the recent cultural invention of mathematics [28,75,76]. It would be very interesting to conduct similar experiments in congenitally blind children, using both spatial-tactile and serial-auditory displays, to see how important vision really is, and if its role can be substituted in those who have never experienced vision.

## 6. Concluding remarks

The human psychophysical results presented here clearly reinforce the animal studies demonstrating the existence of a system dedicated to the perception of numerosity, the number of objects within a particular field of view. This system seems to be quite independent of mechanisms dedicated to texture perception, but is closely connected with systems for estimating the numerosity of sequences of events, in any sensory modality, and also with the production of actions.

Although much work clearly points to the existence of a specialized number sense, it is important to note that this does not preclude the possibility of interactions with other related attributes. In the study of Cicchini *et al*. [46], there were very clear interactions between those attributes when subjects were asked to make explicit subjective judgements about numerosity, density or area. In particular, density judgements were strongly drawn towards number, showing the two are not perceptually independent: but it is density that is primarily influenced by numerosity, rather than the other way round. Interactions between seemingly unrelated perceptual attributes abound: apparent speed depends on luminance, contrast and colour [77,78]; interval duration depends on speed [79–81]; event duration depends on size [82]; number depends not only on size and density, but also on eye movements [83,84], and the region of visual space where stimuli are displayed [85,86]. Specialized systems frequently interact with each other, but this does not preclude their existence. That duration depends on speed and size does equate time with space; and very few would deny the existence of dedicated motion mechanisms, despite clear interactions with luminance, contrast, colour, form and time [87,88]. Similarly, the oft-reported interactions between numerosity, density and area [7,8,89,90] do not preclude the existence of numerosity mechanisms, they merely show how strongly the various mechanisms of our brain are interconnected and interrelated. The strong interconnections between mechanisms estimating magnitude of various quite different dimensions, including space, time and number, are at the heart of the ATOM (a theory of magnitude) theory [91], for which we have also provided supporting evidence [83]. However, the strong evidence for the spontaneous emergence of number suggests that it has a special status within a more general system of magnitude estimation.

Harvey *et al*. [92] have recently reported a clear neuronal map for numerosity, robust to changes in low-level features. These maps cannot be accurately predicted by models of responses based on simple non-numerical visual features, rather than numerosity [93]. Harvey *et al*. have also shown that the same area contains a tuning map for size [94]. The two maps overlap, coexisting in the same cortical area, highly consistent with the notion of a specific brain area dedicated to magnitude estimation [91]. However, the mapping for the two properties is clearly separable: this would allow the independent estimation of size and number, but with a certain amount of cross-talk.

As the density of items increases to a point where they are not individually segregable, numerosity mechanisms fail, and other texture-like mechanisms come into play. We [37] argue that the inability to segment in order to enumerate items may be akin to the phenomenon of *crowding* [58], important for many perceptual tasks, such as reading. As with classical crowding, the transition between mechanisms depends strongly on eccentricity, and is independent of item size.

The idea of multiple systems may account for some seemingly conflicting results reported by different groups, all using non-standardized stimuli. Many different studies have used different stimulus densities, presumably stimulating different mechanisms. For example, the test stimuli used by our laboratory to study numerosity in general fall well within the numerosity regime (unless we are studying texture), under 0.3 dots deg^−2^ and within 15° of the fovea. On the other hand, other studies [7,9] have used stimuli that clearly stimulate the texture region, up to five times the switching density [37, tbl. 1]. Future work should try to standardize on stimulus densities to reduce this confusion in the literature.

To conclude, a great deal of evidence suggests that humans perceive number spontaneously, with dedicated mechanisms. Whether these mechanisms are innate is harder to prove. However, developmental studies show that thresholds for numerosity discrimination are more adult-like at 6 years of age than are those for dense-texture discrimination [38], reinforcing other studies [95] reporting early emergence of number discrimination. Importantly, precision for numerosity, but not texture density, correlates with mathematics achievement in school-age children [38], adding weight to the idea that numerosity mechanisms act as a ‘start-up tool’ for later acquisition of mathematics [68]. This strong link with mathematics provides a further motivation to understand fully the mechanisms underlying the perception of number and, possibly, the foundations for mathematics.

## Data accessibility

This article has no additional data.
